# A Stability Indicating Capillary Electrophoresis Method for Analysis of Buserelin 

**Published:** 2014

**Authors:** Elnaz Tamizi, Ernst Kenndler, Abolghasem Jouyban

**Affiliations:** a Student Research Committee and Faculty of Pharmacy, Tabriz University of Medical Sciences, Tabriz, Iran.; b Institute for Analytical Chemistry, Faculty of Chemistry, University of Vienna, A 1090 Vienna. Waehringerstrase 38, Vienna, Austria.; c Drug Applied Research Center and Faculty of Pharmacy, Tabriz University of Medical Sciences, Tabriz, Iran.

**Keywords:** Buserelin, Capillary zone electrophoresis, Force degradation, Pharmaceutical product, Stability

## Abstract

A simple and rapid stability indicating method based on capillary zone electrophoresis has been developed and validated for the analysis of buserelin (BUS). The best separations were achieved by using a bare fused silica capillary (75 μm *i.d*.; 65.5 cm total, 57.0 cm effective length), phosphate buffer (pH = 3.00; 26.4 mM), at 35 °C. The sample was hydrodynamically injected at 50 mbar for 5 seconds; the applied voltage was 30 kV and detection was carried out by UV-absorbance at 200 nm. Method validation resulted in the following figures of merit : the method was linear in the concentration range between 0.781 and 500 μg/mL (linear regression coefficient 0.9996), accuracy was between 99.3% and 100.9%, intra assay precision was between 0.3% and 1.0% and intermediate precision was between 1.0% and 2.1%. Evaluation of the specificity of the method showed no interference between excipients or products of force degradation and BUS. Under the selected conditions, separation of BUS and its degradation products was completed in less than 10 min, and BUS could be quantified after different stress conditions without any interference. The results enabled the conclusion that under thermal stress upon exposure to 90 °C BUS is degraded by first order kinetics. It was demonstrated that the method can be applied as a rapid and easy to use method for quantification and stability testing of BUS in biopharmaceutical formulations in quality control laboratories.

## Introduction

Due to the extreme improvements in biotechnological methods, such as DNA recombinant technology, in recent years more than 100 proteins have been produced and approved as therapeutic agents for the treatment of different kinds of diseases. According to the development in production of these proteins, there is a growing demand to introduce appropriate analytical methods which could be used for the assessment of their quality in terms of identity, quantity, purity and stability (1, 2). 

Buserelin (BUS) is one of the therapeutic proteins; it is a peptide hormone and consists of 9 amino acids (Pyr–His–Trp–L-Ser–Tyr–D-Ser(tBu)–Leu–Arg–Pro-NH-Et). This peptide is a highly active synthetic analogue of the luteinizing hormone-releasing hormone (LHRH) and acts as an agonist on gonadotropin-releasing hormone (GnRH) receptors. It suppresses the release of both luteinizing hormone (LH) and follicle stimulating hormone (FSH) from the pituitary gland, after an initial increase in secretion of the gonadotropines; thus it could be used in the treatment of hormone responsive cancers such as prostate cancer, breast cancer, endometriosis, uterine leiomyoma, fibroids and in assisted reproduction (3-5). 

Different methods, such as reverse phase HPLC, LC-MS and FAB-MS-MS have been reported for the analysis of BUS (6-9). One of the powerful techniques in protein separation and characterization is capillary electrophoresis (CE), which has been applied for the analysis of different pharmaceutical proteins (1, 10-14). Using these CE methods, BUS is identified and quantified in pharmaceutical and biological samples (15-21). In all cases an acidic background electrolyte (BGE) has been used to charge the analyte, as it has no acidic group in the molecule, but a number of chargeable nitrogen-containing entities (in addition to amid groups, which are very weak bases and can hardly be protonized at the pH values used in CE). In 1998, Wätzig and Degenhardt developed a CE method for the assessment of the stability of BUS acetate (BUS-Ac) and separation from the side components formed either at long storage or under the influence of γ-radiation applied for sterilization. They used a field-amplified sample injection with low conductivity for increasing the sensitivity (15). Sanz-Nebot *et al. *used a CE method for evaluating the migration behavior of therapeutic peptide hormones in standard solutions (16) and Loden and Amini reported a multi-injection CZE method for speeding up the determination of BUS in a pharmaceutical product using a large matrix peak (from benzyl alcohol) as marker (17). CE was combined with MS for identification and quantification of BUS (18-21). Sanz-Nebot *et al. *developed and validated a CE-TOF-MS method for analysis of therapeutic peptide hormones (18) and compared sheathless and sheath-flow electrospray interfaces (19) in CE-ESI-MS. In both papers, standard solutions of the analytes were used as samples. Stanova *et al. *developed and validated a CE-MS method for determination of BUS in urine (20), and reported a CE-ESI-MS method for analysis of therapeutic peptides using preparative isotachophoresis for sample pretreatment (21). It is obvious that these methods need sophisticated instrumentation and skilled personals.

Stability studies are the most critical part in quality control of biopharmaceuticals. As only real time - real condition stability data could indicate the expiration date and suitable conditions for storage and transportation of biopharmaceuticals, stability studies under stress conditions are important, too. The results of such studies could be used for the elucidation of degradation pathways and products. In addition, the stability information of a drug could be used for the development of stability indicating methods where the related degradation products are not available to the analyst. Moreover, the results may be useful for the determination of the effect of accidental exposure of the product to undesirable conditions which may happen during its storage (22-26). It was therefore the aim of the present work to develop and validate a simple and rapid CE method for the analysis of BUS with the focus on the capability to monitor the stability of the drug in biopharmaceutical formulations after force-degradation by thermal stress under acidic, neutral and alkaline conditions. 

## Experimental

*Materials*

BUS acetate (BUS-Ac) powder was purchased from Tofigh Daru Pharmecutical Co. (Tehran, Iran); according to the certificate of analysis, 1.00 mg of this powder is equivalent to 0.95 mg BUS. Pharmaceutical formulations of BUS (solutions in vials, Superfact®, Aventis Pharma, Bad Soden, Germany; with nominal BUS concentration of 1.00 mg/mL (1.05 mg/mL BUS-Ac)) were purchased from a local pharmacy store. Orthophosphoric acid (85%), hydrochloridric acid (37%), sodium hydroxide and sodium dihydrogen phosphate (both analytical grade) were purchased from Merck (Darmstadt, Germany); acetonitrile, methanol and ethanol (all HPLC grade) were obtained from Scharlau (Barcelona, Spain). All solutions were prepared from double distilled water (Millipore, Bedford, MA, USA) and were filtered through a 0.2 μm pore size filter (Macherey–Nagel, Germany) before use.

*Instrumentation*

Analyses were performed using a CE instrument (Agilent Technologies 7100, Waldbronn, Germany), equipped with a photodiode array (PDA) detector with detection range from 190 to 600 nm. Instrument control, peak integrations and peak purity calculations were done by Agilent ChemStation^®^ software and p-value calculations were performed by SPSS 11.5 software. Bare fused silica capillaries were purchased from Agilent Technology. A Metrohm^®^ pH meter (Herisau, Switzerland) was used for pH adjustment.

*CE analysis*

At the beginning of each working day the capillary was flushed for 10 min with sodium hydroxide (0.1 N) and 10 min with double distilled water, respectively, then the capillary was conditioned by the BGE solution for 20 min, between each run the capillary was flushed for 5 min with BGE. The samples were injected hydrodynamically for 5 s at 50 mbar.

*Electrolyte preparation*

A stock solution of phosphate electrolyte (200 mM) was prepared by dissolving the accurately weighted amount of sodium dihydrogen phosphate in double distilled water. For preparation of the BGE at different concentrations, the appropriate volume of the stock solution was diluted with double distilled water and its pH was adjusted by orthophosphoric acid (85%) or sodium hydroxide (5 M).

*Force degradation of BUS*

For evaluating the specificity and stability indicating capability of the method samples were force-degraded. Although ICH Q1A (R2) guideline (27) provides some recommendations about the stability testing of pharmaceutical products, there is no well established protocol for force degradation of biopharmaceuticals. Note that ICH Q5C guideline (26) recommends that stress conditions for biotechnological/biological products “should be selected on a case-by-case basis”; for this reason we choose the stress conditions according to the kind of the analyte and previously published papers (6, 12, 28).

The most probable degradation mechanisms of BUS in aqueous solutions are epimerization and hydrolysis (6). As these reactions depend on the pH, force degradation was carried out at neutral, acidic and basic conditions. Thermal stress was used to accelerate degradation; other samples were exposed to sun light.

The specificity of the method was evaluated with force degraded solutions of pure BUS-Ac with concentration of 1.00 mg/mL (equivalent to 0.95 mg/mL of BUS), which were analyzed (see below), and the peak purity factors using a PDA detector and ChemStation^®^ software were calculated. 

For force degradation at neural solution under thermal stress, 10.00 mg of BUS-Ac powder was dissolved in 10.00 mL of double distilled water and exposed to 90 ± 1 °C for 1000 hours. After defined time intervals, aliquots were diluted 10 fold with double distilled water and analyzed.

For force-degradation under acidic and alkaline conditions, 10.00 mg of BUS-Ac powder was dissolved in 10.00 mL of 0.1 M hydrochloric acid and 0.1 M sodium hydroxide, respectively. One half of the solution was kept at 70 ± 1 °C for 2 hours, the other half was stored at 30 ± 1 °C for 48 hours. 

For evaluating the effect of the sun light on the stability of BUS, the solution of 1.00 mg/mL BUS-Ac in double distilled water and a formulation were subjected to sun light exposure for 48 hours.

*Method development*

Method development was carried out using the initial BUS-Ac stock solution with concentration of 1.00 mg/mL in double distilled water and the force-degraded BUS samples. Fused silica capillaries of 75 μm *i.d*. (with total length of 40.5, 50.5, 65.5, 80.5 cm), or with 50 μm *i.d*. (with total length of 65.5 cm) were used for CE separations; in all cases the detection window was located at 8.5 cm from the capillary end. Buffer solutions consisted of 25, 50 and 75 mM sodium dihydrogene phosphate with pH of 2.00, 3.00, 3.50 and 4.00, respectively. Analyses were run using normal polarity (anode at injector, cathode at detector side) with applied voltages of 20, 22.5, 25, 27.5 and 30 kV at constant temperatures of 15, 20, 25, 30, 35 and 40 °C. Organic modifiers (methanol, ethanol and acetonitrile) were added in the concentration range between up to 5% v/v.

*Method validation*

Validation of the finally selected method was carried out in terms of linearity, accuracy, precision, LOD and LOQ according to ICH (29). The linearity of the method was investigated using the peak areas at BUS-Ac concentrations of 0.781, 1.15, 3.12, 6.25, 12.5, 25.0, 50.0, 100, 250 and 500 μg/mL, from samples which were serially diluted from stock solutions and were analyzed (n = 3 at each concentration). LOQ and LOD were calculated using the slope and standard deviation of the intercepts of three independent calibration curves. The intra assay and intermediate precision and the accuracy of the method were determined at 3 concentrations at the lower, the middle and the upper levels of the calibration curve (at 1.15, 50.0 and 500 μg/mL BUS-Ac). Precision was derived from the calculated amounts for each concentration with the peak area and the calibration equation in repeated analyses (n = 3). For accuracy determination, samples with a known concentration of 1.15, 50.0 and 500 μg/mL BUS-Ac were analyzed in triplicate, then the experimentally derived concentrations were calculated from the peak area and calibration equation. The accuracy at each concentration was reported as percentage of the experimentally derived concentration to the nominal concentration. The specificity of the method was investigated by determination of the peak purity parameters of BUS peaks in standard and real samples (commercial formulations) and also in presence of the degradation products, using a PDA detector and Chem Station^®^ software. For evaluating the robustness of the method, the one-variable-at-time (OVAT) procedure (30) was used. According to OVAT at each step only one factor among those which affect the separation (time of injection, temperature, voltage, pH and concentration of the BGE), were varied in the narrow range around the established conditions while the others were held constant. Then the relative standard deviation (RSD %) and the p*-*value between the results of each step were calculated. The stability of the method was investigated in terms of short term temperature stability, stock solution stability and freeze and thaw stability based on FDA guideline (31). 

*Analysis of formulations*

The ability of the method for the determination of BUS-Ac in pharmaceutical formulations was tested on 3 vials from different batches of a commercial product (Superfact^®^). For analysis, each formulation was diluted with double distilled water to obtain a nominal BUS-Ac concentration of 105 μg/mL, then the samples were analyzed in triplicate by CE and their concentrations were calculated by the use of the calibration equation. Then the assayed amounts were calculated as a percentage of the label claim (nominal concentration).

## Results and Discussion

*Selection of CE conditions*

Absorbance of BUS was recorded in the range of 190 to 400 nm; 200 nm was selected for detection because of the highest UV absorbance at this wavelength. Analyses were finally carried out with a bare fused silica capillary of 75 μm *i.d*., because it gave higher sensitivity than the 50 μm *i.d*. capillary. The capillary with total length of 65.5 cm was chosen for further analysis; although the shorter capillaries gave shorter migration time, increased current resulted which frequently broke down. 

**Figure 1:**
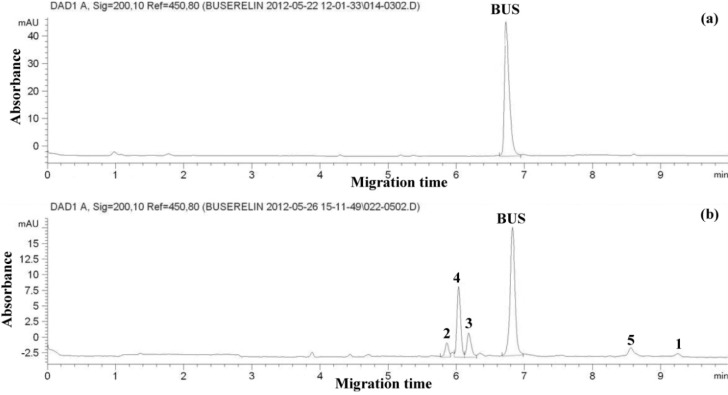
Electropherogram of (a) standard solution of buserelin; (b) thermally degraded neutral solution (a solution of BUS-Ac in double distilled water exposed to thermal stress at 90 °C for 300 hours). Separation conditions: bare fused silica capillary, 75 μm *i.d*.; 65.5 cm length, (effective length = 57.0 cm), BGE, sodium phosphate pH = 3.00 (26.4 mM phosphate); cassette temperature 35 °C; applied voltage 30 kV; hydrodynamic injection at 50 mbar for 5 s; detection: UV absorbance at 200 nm.

Suitable separation of BUS and degradation products was achieved by normal polarity, constant voltage of 30 kV, and 35 °C; it was applied for analysis due to the shorter migration times and higher plate numbers. Variation of pH and concentration of the BGE gave most favorable results (short migration time, better peak shapes and resolution, low current) with phosphate buffer (26.4 mM) at pH 3.00 (it consisted of 1000 mL sodium dihydrogen phosphate (25 mM) and 0.1 mL of orthophosphoric acid (85%)). No improvement in the separation was obtained upon addition of organic modifiers. Samples were finally injected by hydrodynamic injection at 50 mbar for 5 seconds. An electropherogram of BUS-Ac standard solution (50 μg/mL) and a thermally degraded sample (a solution of BUS-Ac in double distilled water keeping at 90 °C for 300 hours) under these conditions is shown in Figure 1. It can be seen that the separation of the analyte from its 5 degradation products can be completed within less than 10 min. 

*Validation of the CE method*

**Figure 2:**
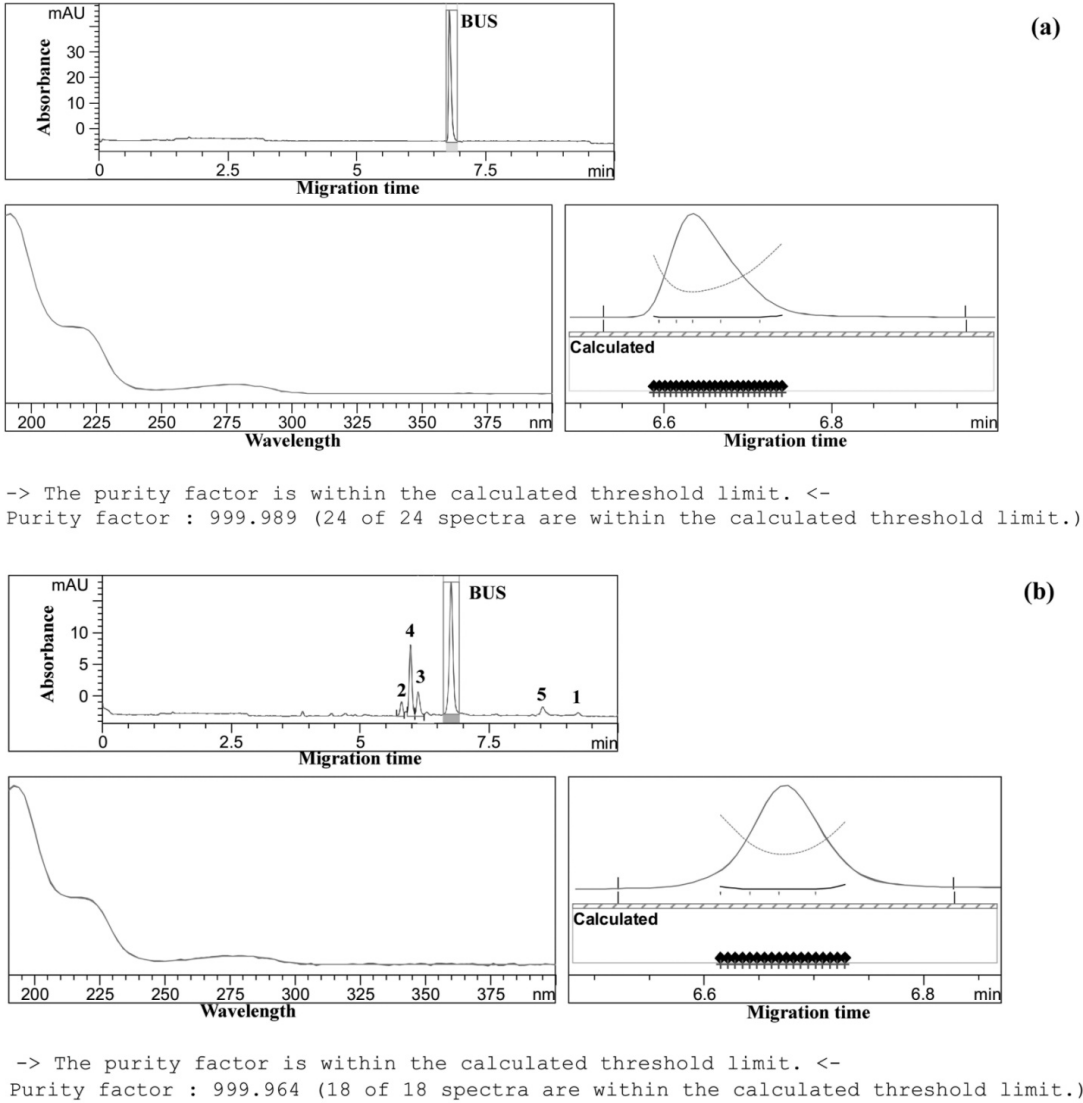
Peak purity evaluation by ChemStation^®^ software. (a) peak purity of BUS in pharmaceutical dosage form, (b): peak purity of BUS in force degraded sample (under thermal stress upon exposure of the neutral sample solution (a solution of BUS-Ac in double distilled water) to 90 °C for 300 h). Separation conditions as in Figure 1.

The specificity of the method was evaluated by the aid of the peak purity factors and the similarity/threshold ratios of BUS peaks in standard solutions, in formulations and in force-degraded samples. As a result, peak purity factors in all samples were between 999 and 1000, similarity/threshold ratios were smaller than 1; the proposed method was thus sufficiently specific for the determination of BUS (32), and possible exicipients or degradation products do not interfere the determination of the drug (Figure 2). 

The method was linear in the investigated range of 0.781 to 500 μg/mL BUS-Ac with a correlation coefficient of 0.9996, the according calibration equation was y = 2633x + 0.017, with x being the concentration of BUS-Ac in micro g/mL and y the peak area in mAU.s. LOQ and LOD were calculated by the standard deviation of the intercept (s = 0.21, n = 3) and the slope of calibration curve, and were 0.78 and 0.39 μg/ mL, respectively. Accuracy and precision of the method are reported in Table 1. According to the results, the present method is accurate and precise in all concentrations which meet the guideline on validation of analytical methods by ICH (29). 

**Table 1:** Accuracy and precision of the method

**Conc. BUS-Ac (μg/ml)**	**Accuracy ± SD ** a **(%)**	**Intra-assay precision (RSD ** b **%)**	**Intermediate precision (RSD %)**
1.56	99.8 ± 1.0	1.0	2.1
50.0	99.7 ± 0.5	0.5	1.6
500	100.9 ± 0.3	0.3	1.0

a SD = standard deviation.

b RSD = relative standard deviation

The results for the method stability (derived according the FDA rules) are reported in Table 2. According to the data, BUS samples were stable under different conditions: the recovery is larger than 98% in all cases. 

**Table 2:** Results of method stability at different temperature conditions

**Stability Conditions**	**Recovery ± SD (%)**
Room temperature/4 h	99.8 ± 0.7
4 °C / 48 h	99.3 ± 0.7
3 Freeze-thaw cycles (-20 °C / +25°C)	98.2 ± 1.1

The results presented in Table 3 show that the method was robust because the relative standard deviation of the results obtained from the OVAT procedure were smaller than 2%; the analysis of the results by ANOVA showed that the difference their differences are statistically not significant (p-value > 0.05). 

**Table 3:** Results of method robustness with the one-variable-at-time (OVAT) method

**Variable**	**Variation range**	**Assay (%)** a	**RSD%**	**p-value**	**Migration time (min)**	**RSD%**	**p** ***-*** **value**
Injection time (s)	4	98.0	1.6	0.42	6.98	0.2	0.48
	5	99.8	0.7		6.97	0.3	
	6	100.0	1.0		6.90	1.0	
Temperature (°C)	33	98.2	1.0	0.47	7.72	1.1	0.13
	35	99.4	0.6		6.87	0.2	
	37	99.3	1.6		6.20	1.1	
Buffer pH	2.98	98.3	1.4	0.28	7.04	1.0	0.46
	3.00	99.9	0.7		6.89	1.1	
	3.02	101.3	0.9		7.12	1.6	
Buffer concentration (mM)	24.4	98.4	1.6	0.52	6.95	0.5	0.60
	26.4	100.0	0.5		6.89	1.4	
	28.4	100.5	1.3		6.78	0.8	
Voltage (kV)	29	99.3	1.0	0.58	7.07	1.6	0.55
	30	100.0	0.5		6.84	0.9	

a At each step the same sample (100 µg/ml BUS-Ac) was analyzed 3 times, and average of obtained concentrations and migration times are shown.

Details of the analytical performance of previously reported and the present CE methods for the quantification of BUS are summarized in Table 4. Beside its acceptable accuracy and precision, the present method has a lower LOQ and wider linearity range. 

**Table 4:** Comparison of figures of merit of the present method for determination of buserelin with other CE methods reported in the literature

**Method**	**Linearity range (μg/mL)**	**Correlation coefficient**	**LOD** **(μg/mL)**	**LOQ** **(μg/mL)**	**Accuracy** **(%)**	**Precision** **(RSD%)**	**Repeatability of peak area (RSD%)**	**Ref.**
CE	11.0-400	0.9960	4.0	11	97.5-101.7 a	2.3-4.9 b	1.4	(17)
CE-MS	2.45-98.0	0.9990	0.98	2.4	-	-	11-14	(18)
CE-MS (Sheathless)	-	-	2.5	-	-	-	41	(19)
CE-MS (Sheath-flow)		-	0.98	-	-	-	14	(19)
CE-MS (Pressure injection)	1.4-20	0.9996	0.47	1.4	-		9.7	(20)
CE-MS (Electrokinetic injection)	0.96-20	0.9977	0.32	0.96	-	-	5.4	(20)
CE-MS	1.94-10.0	0.9984	0.64	1.94	-	-	4.2 – 9.1	(21)
CE	0.78-500 (BUS-Ac)	0.9996	0.39	0.78	99.7-100.9	0.3-2.1	0.28-5.4 ^2^	This work

a Accuracy and precision were calculated for 2 standard solutions containing 75 and 100 µg/mL BUS.

b Range for repeatability of peak area was calculated for all concentrations of calibration curve.

*Analysis of formulations and force-degraded samples*

The content of three vials of a commercial formulation was diluted to the final nominal concentration of 105 μg/mL BUS-Ac, and the samples were analyzed by the proposed method. The assayed amounts of BUS-Ac for three samples were 99.8 ± 1.7, 98. 8 ± 0.8 and 99.7 ± 1.9 % (p*-*value = 0.88). 

**Figure 3:**
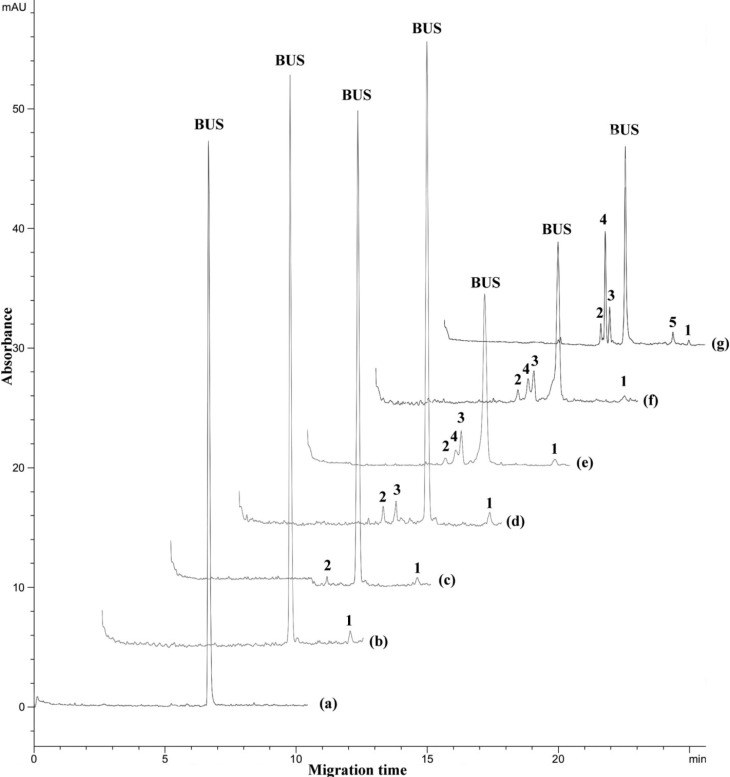
Electropherograms of force degraded samples under different stress conditions: (a) standard solution of buserelin; (b) sun light exposure for 24 h, (c) acidic stress at 70 °C for 2 h; (d) acidic stress at 30 °C for 24 h; (e) alkaline stress at 70 °C for 2 hours; (f) alkaline stress at 30 °C for 24 h; (g) thermal stress at 90 °C in a neutral solution (a solution of BUS-Ac in double distilled water) after 300 h. Peaks 1, 2, 3, 4 and 5 indicate the degraded forms. Separation conditions as in Figure 1.

In Figure 3, electropherograms of force-degraded samples are shown; it can be seen that, under all conditions the BUS peak decreases, pointing to the decomposition of the drug. Only one degradation product is formed under photolytic stress (peak 1, trace b). The most pronounced degradation is observed at neutral solution under thermal stress upon exposure of the sample to 90 °C for 300 hours (trace g). The effect is comparably small when the sample is exposed to 70 °C for 2 hours in an acidic solution: here only 2 small peaks (numbers 1 and 2) evolved. The effect of the acidic environment is more pronounced when the sample is kept at 30 °C temperature for 24 hours (trace c): in addition the third peak (number 3) arises. Alkaline hydrolysis under these conditions leads to a much stronger degradation of BUS: its peak is much smaller, and an additional product (peak 4) is formed; moreover, the peaks of the decomposition products are much larger in relation to the BUS peak compared to those formed in the acidic environment. This product (peak 4) is most pronouncedly created at neutral solution upon thermal stress at 90 °C (trace g), and an additional product (peak 5) is arising as well. However, quantification is hardly possible even given that the decomposition is caused by a controlled hydrolysis. As can be seen from the structure of the peptide and according to the previous work (6), the most probable degradation reactions are including breakage of the peptide bond on the N-terminal side of the L-Ser residue, elimination of the *tert*-butyl moiety of the D-Ser-(tBu) residue and formation of diketopiperazine or cyclo-(His-Trp). These reactions and the other possible hydrolysis steps can result in products of different amount of UV-absorbing groups (aromatic rings, C=O, other double bonds, *etc*.). Therefore the individual decay products may have different absorption coefficients and exhibit a different response to the UV detector. 

**Figure 4:**
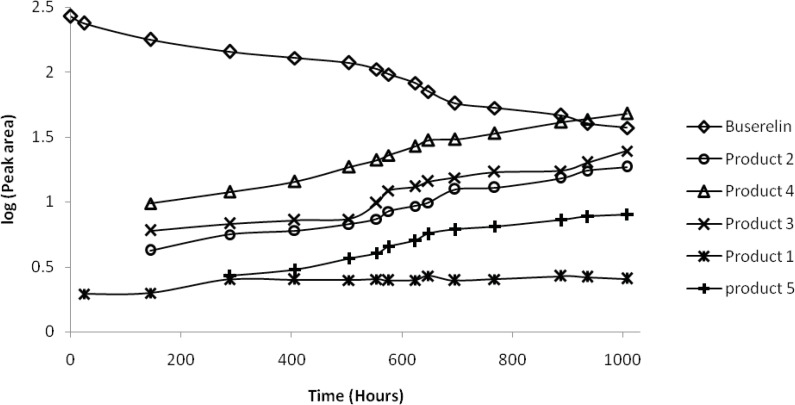
Peak area of buserelin and degraded forms in logarithmic scale as function of time under thermal stress at 90 °C in a neutral solution (a solution of BUS-Ac in double distilled water). Degradation products were numbered according to Figure 1.

According to the migration times and peak shapes of degradation products under different stress conditions (Figure 4), it could be claimed that the same product with a minor peak (product 1) was formed under different stress conditions, the same products 2 and 3 were formed at acidic, basic and thermal stress and product 4 was formed at basic and thermal stress conditions. If necessary, more identification studies are required to approve these claims. 

**Figure 5:**
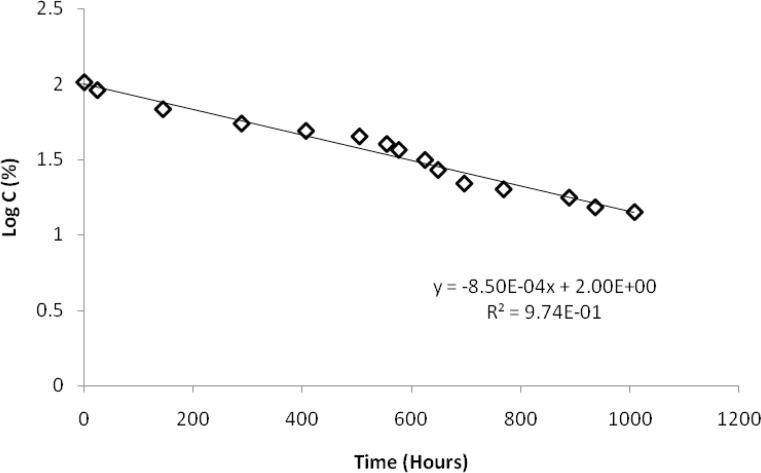
Correlation between logarithm of buserelin concentration and time under thermal stress at 90 °C in a neutral solution (a solution of BUS-Ac in double distilled water).

A closer view to the degradation process caused by thermal stress at 90 °C allows getting insight into the kinetics of the decay. For this purpose, the peak areas were measured during 1000 hours as a function of time of exposure. Peak area of BUS decreased with time, and an increase of the peak areas of products 2, 3 and especially 4 was observed, whereas that of the product 5 increased slowly, and that of product 1 remained approximately constant. These changes are depicted on a logarithmic scale in Figure 4. It can be seen from Figure 5, that a linear relationship between the logarithm of the concentration of BUS and the stress time is observed, with the correlation coefficient of 0.987. This finding indicates that the degradation of BUS-Ac under thermal stress is governed by a first order kinetic according to the following:

Equation(1) $$\log C=\log C_{0}-\left(\frac{K_{90}}{2.303}\right)t=2.002-0.00085t$$

Here *C *is concentration of BUS-Ac in percentage, C_0_ is the initial concentration (100%) at time zero, and *t *is time of degradation in hours; *k*_90 _is the rate constant of degradation of BUS-Ac at 90 °C; its value is 0.00196 h^-1^. 

## Conclusion

We have applied a CZE method to separate BUS from products formed by force degradation mainly under thermal stress in different chemical environments (neutral, acidic and alkaline). The method was validated and was linear (r = 0.9996) in the investigated concentration range of BUS-Ac; accuracy, precision and robustness meets the demands of pharmaceutical analysis; LOD and LOQ were 0.39 μg/mL and 0.78 μg/mL, respectively. BUS could be separated from the five degradation products detected within 10 minutes without need for organic modifiers. In comparison with previous papers (17-21) the present method exhibits a wider linearity range and a lower LOQ. The method has stability indicating capability: BUS can be quantified in presence of the degradation products without any interference; it is robust according to the OVAT procedure (30). The method was successfully used for determination of BUS-Ac content in a pharmaceutical product (assay = 99.4% (s = 1.8, n = 3). In addition, the method enables the investigation of the degradation kinetic of BUS under thermal stress. It follows that the method could be applied for quantification of BUS also as a rapid, simple and robust stability indication method for monitoring the quantity, quality and stability of BUS in the bulk and finished biopharmaceutical formulations in quality control laboratories. 
